# Enhanced splicing modulation by NMA-modified antisense oligonucleotides

**DOI:** 10.1093/nar/gkag484

**Published:** 2026-05-21

**Authors:** Karen Ling, Thazha P Prakash, Jinghua Yu, Michaela Jackson, Seung J Chun, Gemma Bachmann, Noah Post, Sarah Greenlee, Armand Soriano, John L Hunyara, Daniel A Norris, Paymaan Jafar-nejad, Eric E Swayze, C Frank Bennett, Frank Rigo

**Affiliations:** Ionis Pharmaceuticals Inc., 2855 Gazelle Ct., Carlsbad, CA 92010, United States; Ionis Pharmaceuticals Inc., 2855 Gazelle Ct., Carlsbad, CA 92010, United States; Ionis Pharmaceuticals Inc., 2855 Gazelle Ct., Carlsbad, CA 92010, United States; Ionis Pharmaceuticals Inc., 2855 Gazelle Ct., Carlsbad, CA 92010, United States; Ionis Pharmaceuticals Inc., 2855 Gazelle Ct., Carlsbad, CA 92010, United States; Ionis Pharmaceuticals Inc., 2855 Gazelle Ct., Carlsbad, CA 92010, United States; Ionis Pharmaceuticals Inc., 2855 Gazelle Ct., Carlsbad, CA 92010, United States; Ionis Pharmaceuticals Inc., 2855 Gazelle Ct., Carlsbad, CA 92010, United States; Ionis Pharmaceuticals Inc., 2855 Gazelle Ct., Carlsbad, CA 92010, United States; Ionis Pharmaceuticals Inc., 2855 Gazelle Ct., Carlsbad, CA 92010, United States; Ionis Pharmaceuticals Inc., 2855 Gazelle Ct., Carlsbad, CA 92010, United States; Ionis Pharmaceuticals Inc., 2855 Gazelle Ct., Carlsbad, CA 92010, United States; Ionis Pharmaceuticals Inc., 2855 Gazelle Ct., Carlsbad, CA 92010, United States; Ionis Pharmaceuticals Inc., 2855 Gazelle Ct., Carlsbad, CA 92010, United States; Ionis Pharmaceuticals Inc., 2855 Gazelle Ct., Carlsbad, CA 92010, United States

## Abstract

Aberrant RNA splicing contributes to many human diseases, and splice-switching antisense oligonucleotides are ideally suited as a therapeutic strategy to modulate splicing and restore normal gene expression. Nusinersen (Spinraza™) has revolutionized the treatment of spinal muscular atrophy. It is a splice-switching oligonucleotide (SSO) modified with 2′-*O*-methoxyethyl (MOE). Here, we evaluate a next-generation ribose modification, 2′-*O*-[2-(methylamino)-2-oxoethyl] (NMA), which enhances the pharmacological properties of SSOs. We identified a long-lasting NMA-modified human candidate SSO, salanersen, that is three to four-fold more potent than nusinersen in human *SMN2* transgenic mice. To evaluate the generality of the NMA chemistry, we applied it to modulation of *SCN1A* exon 20N splicing, a therapeutic strategy for Dravet syndrome. An NMA-modified SSO is 3.5 -fold more potent than STK-001, a MOE-modified SSO currently in clinical trials. Our data establish the NMA chemistry as a broadly applicable ribose modification that markedly improves the pharmacological profile of SSOs, supporting its development as a next-generation platform for splicing modulation therapies.

## Graphical Abstract

**Graphical Abstract:**
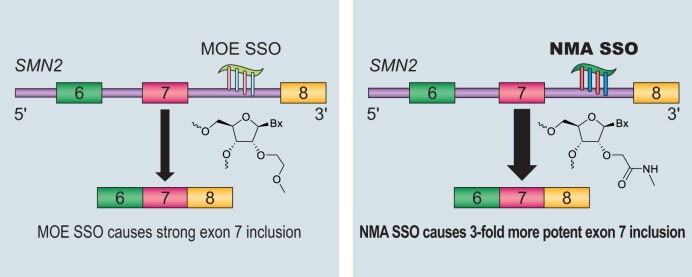

## Introduction

Alternative splicing is ubiquitous in eukaryotic cells and expands the proteomic output of the genome [1, 2]. Mutations in *cis*-acting splicing elements or in *trans*-acting splicing factors that disrupt splicing frequently contribute to many human diseases. Splice-switching oligonucleotides (SSOs) have emerged as a therapeutic strategy to correct splicing defects or modulate gene expression by redirecting splicing [3]. SSOs are short (15–25 nt) antisense oligonucleotides that are chemically modified throughout their length. They hybridize to pre-mRNA and redirect splicing by sterically blocking splicing factors. SSOs have been used successfully to modulate splicing for many targets in a variety of cell types and tissues and many SSOs have advanced into individualized N-of-1 studies as well as broader clinical trials [4–9]. Furthermore, several SSOs have received marketing authorization [10–15]. In the central nervous system (CNS), two diseases where SSOs have advanced the furthest are spinal muscular atrophy (SMA) and Dravet syndrome.

SMA is a hereditary neurodegenerative disease characterized by the loss of motor neurons, progressive muscle weakness, and atrophy [16]. This condition is caused by homozygous loss-of-function mutations in the *SMN1* gene, leading to a deficiency of the SMN protein [17, 18]. Although the paralogous gene *SMN2* also produces the SMN protein, its transcripts yield only 5%–10% of the functional, full-length protein. The limited production of functional protein from *SMN2* is largely due to a C-to-T transition that disrupts splicing regulation, promotes exon 7 skipping, and results in a truncated, nonfunctional protein [19–21]. Nusinersen (Spinraza™) is a 2′-*O*-methoxyethyl (MOE)-modified SSO that binds ISS-N1, an intronic splicing silencer in intron 7 of *SMN2* pre-mRNA [22]. By masking ISS-N1, nusinersen prevents the binding of splicing repressors (notably hnRNP A1/A2), thereby promoting exon 7 inclusion and yielding full-length SMN protein [23]. Nusinersen demonstrated strong positive results in two randomized, sham-controlled trials in patients with SMA [10, 11]. In infants with devastating early-onset disease, treatment markedly increased survival without permanent ventilation and substantially improved motor milestone attainment [10]. In children with later-onset SMA, nusinersen led to robust improvements in overall motor function, while untreated patients experienced progressive decline [11].

The success of nusinersen has propelled efforts to test SSOs in additional CNS diseases, including Dravet syndrome, which is a severe developmental and epileptic encephalopathy most often caused by heterozygous loss-of-function mutations in the *SCN1A* gene, resulting in haploinsufficiency of the voltage-gated sodium channel Nav1.1. The *SCN1A* gene produces an abundant splice isoform that includes a poison exon known as exon 20N. Inclusion of exon 20N introduces a premature termination codon, leading to transcript degradation through nonsense-mediated decay (NMD) [24, 25]. Modulating splicing to reduce inclusion of exon 20N in transcripts from the functional *SCN1A* allele prevents NMD, thereby increasing the levels of *SCN1A* mRNA (messenger RNA) and Nav1.1 protein. Preclinical studies in a mouse model of Dravet syndrome have demonstrated that SSOs designed to exclude exon 20N increase the levels of Nav1.1 protein, reduce seizures, and extend survival [26, 27]. Based on these findings, an MOE-modified SSO (STK-001) has advanced into clinical evaluation in Dravet syndrome patients, with promising early results (ClinicalTrials.gov ID: NCT04740476) [28].

In mouse models of SMA, several types of SSOs have been tested, including those based on 2′-*O*-methyl (2′-OMe), phosphorodiamidate morpholino oligomers (PMOs), and MOE chemistries. When administered to the CNS, SSOs with these chemistries promote exon 7 inclusion, increase SMN protein expression, and extend survival [29–33]. In comparative dose response studies in *SMN2* transgenic mice, MOE SSOs were more potent at promoting exon 7 inclusion than PMO or 2′-OMe SSOs. Constrained ethyl–modified oligonucleotides, evaluated in *SMN2* transgenic mice, showed activity but no clear advantage over MOE [34]. By contrast, an SSO with 2′-fluoro modifications unexpectedly induced *SMN2* exon 7 skipping by recruiting the RNA-binding proteins ILF2/ILF3 [35]. Additional chemistries, such as tricyclo-DNA have also shown activity in mice [36], but MOE remains the chemistry with the most advanced clinical progress for splicing modulation. Thus, it is the only chemistry that has been explored for modulating *SCN1A* exon 20N splicing for the treatment of Dravet syndrome [26].

Despite the success of 2′-MOE-modified SSOs for CNS disease, advances in oligonucleotide chemistries provide an opportunity to further enhance the profile of SSOs. More potent SSOs should result in better clinical response at lower doses, potentially improving their safety profile. In addition, extending the dosing interval would be highly desirable for intrathecally administered SSOs. A series of 2′-*O*-[2-(amino)-2-oxoethyl]–modified ASOs have previously been shown to have high binding affinities to complementary RNA, comparable to the MOE modification [37]. Within this class of 2′-*O*-substituted sugar modifications, the 2′-*O*-[2-(methylamino)-2-oxoethyl] (NMA) modification emerged as a distinct analog, displaying structural features reminiscent of MOE in crystallographic analyses, but with superior metabolic stability. We previously demonstrated that the NMA modification significantly improves the binding affinity to complementary RNA and 3′-nuclease stability relative to DNA [37, 38]. Furthermore, RNase H-mediated degradation of mRNA using ASOs modified with NMA or MOE exhibited similar potency both *in vitro* and *in vivo* [39]. However, NMA and its analogs have not been previously investigated in the context of SSOs. Here, we describe the synthesis, optimization, and pharmacological characterization of NMA-modified SSOs targeting *SMN2* and *SCN1A*.

## Materials and methods

### Synthesis of NMA and analogs-modified nucleoside phosphoramidites

NMA-modified nucleoside phosphoramidites (bzA, ibuG, bz5-MeC, and 5-MeU) were synthesized according to the previously reported procedure [39]. The 5-MeU phosphoramidites modified with 2′–*O*–[2–(*N,N*-dimethylamino)–2–oxoethyl] (DMA) (Supplementary Scheme S1), 2′–*O*–[2–(ethylamino)–2–oxoethyl] (NEA) (Supplementary Scheme S2), 2′–*O*–[2–(propylamino)–2–oxoethyl] (NPA) (Supplementary Scheme S3), 2′–*O*–[2–(cyclopropylamino)–2–oxoethyl] (NcPA) (Supplementary Scheme S4), and 2′–*O*–[2–(cyclopropylmethylamino)–2–oxoethyl] (McPA) (Supplementary Scheme S5) were prepared according to the synthetic schemes described in the Supplementary data. Likewise, bz5-MeC phosphoramidites modified with DMA (Supplementary Scheme S6), NEA (Supplementary Scheme S7), NPA (Supplementary Scheme S8), NcPA (Supplementary Scheme S9), and McPA (Supplementary Scheme S10) were synthesized following the corresponding schemes presented in the Supplementary data.

### Oligonucleotide synthesis

The synthesis and purification of the chemically modified SSOs were performed according to previously reported methods [39]. The oligonucleotides were characterized by ion-pair high-performance liquid chromatography–mass spectrometry (ion-pair HPLC–MS) analysis with Agilent 1100 MSD system (Supplementary Table S2). The oligonucleotides targeting human *SMN2* were 2′-*O*-MOE or 2′-*O*-NMA modified; SSOs were either 18 or 20 nucleotides in length. The parent SSO sequence (MOE-1) targeting the human *SMN2* gene was 5′-TCACTTTCATAATGCTGG-3′. The SSO sequence targeting *SCN1A* gene was 5′-AGTTGGAGCAAGATTATC-3′. The lyophilized SSOs were dissolved and diluted to the desired concentration in sterile phosphate-buffered saline (PBS) without calcium or magnesium for experiments in mice. The SSO was quantified by UV spectrometry and sterilized by passage through a 0.2-µm filter before dosing.

### Intracerebroventricular administration of SSO in mice

Animal housing and all procedures met ethical standards for animal experimentation and were approved by the Institutional Animal Care and Use Committee at Ionis Pharmaceuticals. Adult male and female SMA Type III mice [FVB.Cg-*Smn1^tm1Hung^* Tg(SMN2)2Hung/J, stock number 005058] were obtained from The Jackson Laboratory (Bar Harbor, ME). Mice were anesthetized and placed on a stereotaxic frame fitted with a nose cone continuously supplied with 2% isoflurane. The scalp and anterior back were shaved and disinfected. An ∼1-cm incision was made on the scalp, and the subcutaneous tissues and periosteum were scraped from the skull with a sterile cotton-tipped applicator. A 10-μl Hamilton microsyringe with a 26G Huber point removable needle was driven through the skull at 0.2 mm posterior and 1.0 mm lateral to bregma, and was lowered to a depth of 3 mm. Ten microliters of SSO solution or PBS were administered to the right cerebral ventricle at a rate of 1 µl per second. Three minutes after the injection, the needle was slowly withdrawn and the incision was sutured. The mice were then allowed to recover from anesthesia in their home cage.

### RNA extraction and mRNA analysis

At necropsy, tissues collected were snap frozen at −70°C or below within 5 min and later processed for RNA extraction and mRNA analysis by reverse transcription-quantitative polymerase chain reaction (RT-qPCR). For the mouse spinal cord, a 2-mm lumbar section was collected. For the brain, a 1-mm coronal section, 2 mm posterior to the injection site, was collected. RNA extraction and mRNA analyses were performed as previously described [34]. Briefly, each piece of tissue was homogenized in a 2-ml tube containing Lysing Matrix D (MP Biomedicals, Santa Ana, CA) and 500 µl of RLT buffer (Qiagen, Valencia, CA), and 1% (v/v) β-mercaptoethanol. Homogenization was performed for 20 s at 6000 rpm using a FastPrep Automated Homogenizer (MP Biomedicals). Total RNA was extracted from 10 µl of lysate using a RNeasy 96 Kit (Qiagen), which included on-column DNase I digestion (50 U; Invitrogen, Carlsbad, CA). One-step RT-qPCR was performed on the StepOne Real-Time PCR systems with the EXPRESS One-Step SuperScript™ RT-PCR kit (Thermo Fisher Scientific, Carlsbad, CA) using gene-specific primers as described previously [34, 35]. Primer and probe sequences are listed in Supplementary Table S3. Each 30-µl reaction consisted of 15 µl of 2X EXPRESS SuperScript™ Mix, 300 nM of each forward and reverse primer, 100 nM probe, 0.6 µl ROX reference dye, 10 µl corresponding to 2–4 ng total RNA, and nuclease-free water. Reverse transcription was carried out at 50°C for 15 min, followed by an initial denaturation at 95°C for 2 min and 45 cycles of amplification consisting of denaturation at 95°C for 15 s, annealing/extension at 60°C for 1 min. No-RT controls were included to confirm the absence of genomic DNA contamination, and no-template controls were included for each primer probe set to assess potential contamination or nonspecific amplification. All reactions were performed in technical triplicates, and at least three independent biological replicates were analyzed per experimental condition. RT-qPCR data were analyzed using StepOne software (v.2). Quantification cycle (Cq) values were determined at a threshold set within the exponential phase of amplification, above baseline and below plateau, with baseline correction applied. Amplification efficiency (*E*) and correlation coefficient (*R*²) for each primer pair were calculated from standard curves generated using four 2.5-fold serial dilutions of 4 ng total RNA. Acceptable efficiency was defined as 90%–110% and *R*² ≥ 0.99 and Cq values ranging between 24 and 29; all primer probe sets met these criteria. For FL or Δ7 *SMN2* transcript quantification, the FL or Δ7 *SMN2* expression level was normalized to that of *Gapdh* or total *SMN2* and further normalized to the level in vehicle (PBS)-treated animals or cells. For the analysis of glial fibrillary protein (*Gfap*), allograft inflammatory factor-1 (*Aif1*), or *Cd68* expression, normalization was to *Gapdh* levels and further normalized to vehicle (PBS)-treated animals. Results are reported as mean ± standard deviation (SD). Statistical analyses between treatment groups were performed as described in the data analysis section.

### Quantification of SSO tissue concentration

A 1-mm mouse brain coronal section, 3 mm posterior to the injection site and the thoracic spinal cord, was collected for bioanalytical evaluation. Each piece of tissue was weighed, and the amount of SSO was then measured by various bioanalytical methods [40], including capillary gel electrophoresis coupled with UV detection, high-performance liquid chromatography (HPLC) coupled with UV detection, HPLC coupled with tandem mass spectrometry detection, or a hybridization-based enzyme-linked immunosorbent assay (HELISA). For HELISA, the probes have a sequence complementary to the SSO. The probe for MOE-1 contained biotin–tetraethylene glycol (TEG) at the 5′ end and digoxigenin at the 3′ end. The probe for NMA-3 contained digoxigenin at the 5′ end and biotin–TEG at the 3′ end.

### Data analysis and statistics

Statistical analyses of transcript levels comparing SSO-treated groups and vehicle-treated control groups were performed using an unpaired two-tailed *t*-test in GraphPad Prism software (version 6.0 or higher; GraphPad Software, San Diego, CA). Dose- and concentration-response relationships were analyzed in GraphPad Prism software version 6.0 or higher (GraphPad Software, San Diego, CA). Data were analyzed by nonlinear regression with a variable–slope (four-parameter) model. For determination of half-maximal response values (ED_50_ for dose-response and EC_50_ for SSO concentration-response), minimal response values were constrained to 1 to improve curve fitting accuracy. ED_50_ and EC_50_ values were calculated from the fitted curves and are reported with 95% confidence intervals (CI). Comparisons of ED_50_ or EC_50_ values between groups were conducted using nonlinear regression with extra sum-of-squares F test. Time course response to SSO treatment were compared using two-way ANOVA with post-hoc Šídák’s multiple comparisons tests for each time point. Statistical significance was defined as *P < .05, **P < .01, ***P < .001, ****P < .0001. Values of *P* ≥ .05 were reported as not significant (ns). The tissue half-life of SSOs associated with the apparent terminal elimination phase was calculated using a noncompartmental analysis (extravascular input model) applied to the mean concentration–time profile using Phoenix WinNonlin (Version 8.3, Certara, L.P., Princeton, NJ).

## Results

### Enhanced potency of an NMA-modified SMN2 SSO

**Figure 1.**
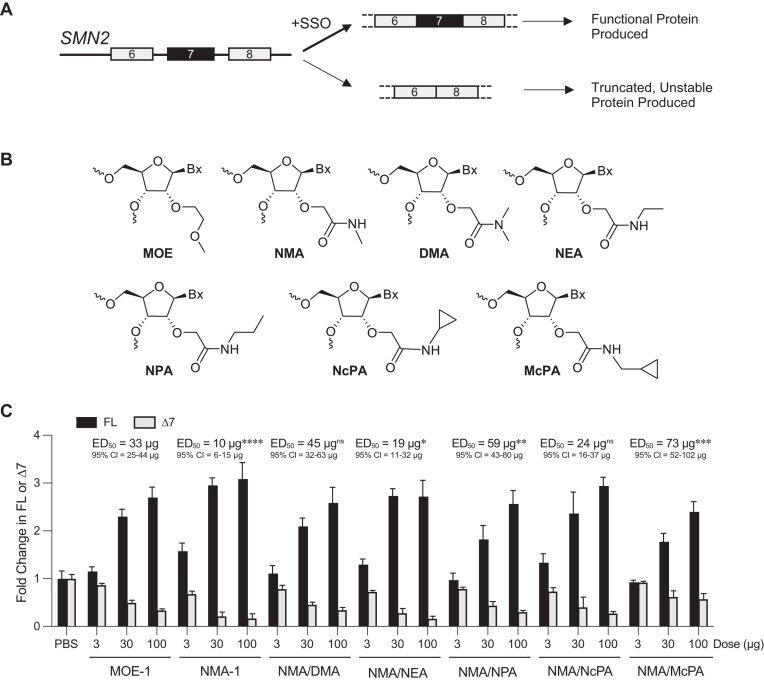
Potency for *SMN2* splicing correction of SSOs modified with MOE, NMA, or NMA analogs in the spinal cord of human *SMN2* transgenic mice. (**A**) Schematic diagram of naturally occurring productive versus nonproductive alternative splicing of *SMN2* pre-mRNA. The *SMN2* transcript lacking exon 7 encodes a truncated SMN protein that is unstable and rapidly degraded. SSO promotes inclusion of exon 7 generating a full-length mRNA and functional protein. (**B**) Chemical structures of nucleotides modified with MOE, NMA, and NMA analogs. (**C**) RT-qPCR analysis of *SMN2* transcripts including exon 7 (FL) or skipping exon 7 (Δ7) in the spinal cord 14 days after ICV bolus injection of MOE-1, NMA-1, or SSOs modified with analogs of NMA in *SMN2* transgenic mice. For each dose level, *N* = 4 per group. Data are mean ± SD. The ED_50_ for exon 7 inclusion (FL) and 95% CI are shown. Comparisons of ED_50_ between MOE-1 and each NMA or NMA-analog SSO were performed by nonlinear regression with an extra sum-of-squares F test. *P < .05, **P < .01, ***P < .001, ****P < .0001; ns, not significant.

Pre-mRNA splicing of the *SMN2* gene predominantly generates a transcript lacking exon 7 and results in an unstable SMN protein that is rapidly degraded (Fig. 1A). Nusinersen, an SSO uniformly modified with MOE, promotes exon 7 inclusion and increases SMN protein levels [34,41]. We set out to identify a chemical modification with improved *in vivo* potency (i.e. greater activity at a lower dose) for *SMN2* exon 7 inclusion compared to nusinersen. A series of SSOs containing NMA or NMA analogs (Fig. 1B) with varying steric and electronic properties were designed and synthesized (Supplementary Table S1). The melting temperatures (*T*_m_) of the SSOs incorporating the novel NMA analogs were comparable to the NMA SSO, with the exception of DMA, which induced a modest destabilization of approximately −0.7°C per modification (Supplementary Table S1). To evaluate the activity of the ASOs *in vivo*, increasing doses of the uniform MOE SSO nusinersen (here referred as MOE-1), a uniform NMA SSO (NMA-1), or SSOs modified with analogs of NMA (Fig. 1B and Supplementary Table S1), were administered to the cerebrospinal fluid (CSF) of adult human *SMN2* transgenic mice [42] via a single intracerebroventricular (ICV) bolus injection. Spinal cord and brain tissues were analyzed for *SMN2* splicing 14 days post-injection by RT-qPCR. All SSOs induced dose-dependent *SMN2* splicing correction, evidenced by an increase in full-length (exon 7–included) transcripts and a corresponding decrease in Δ7 (exon 7–skipped) transcripts in both the spinal cord (Fig. 1C) and the brain (Supplementary Fig. S1). Based on the half-maximal effective dose (ED_50_) for promoting *SMN2* exon 7 inclusion, the NMA-modified SSO (NMA-1) emerged as the most potent, exhibiting the lowest ED_50_ value among all SSOs tested (10 µg versus 33 µg in the spinal cord and 14 µg versus 39 µg in the brain for NMA-1 versus MOE-1, respectively). The improved potency of NMA-1 was also observed in an independent study (Supplementary Fig. S2).

To more precisely compare the potency for *SMN2* splicing correction of the NMA versus MOE-modified SSOs, we performed a five-point dose-response of MOE-1 and NMA-1 for *SMN2* correction in *SMN2* transgenic mice. *SMN2* splicing correction was measured in the spinal cord and the brain harvested 2 weeks after the ICV bolus injection by RT-qPCR. The half-maximal effective dose (ED_50_) for *SMN2* splicing correction by the NMA-1 SSO was 5 µg in the spinal cord and 9 µg in the brain, and the ED_50_ for *SMN2* splicing correction by the MOE-1 SSO was 22 µg in the spinal cord and 32 µg in the brain (Supplementary Fig. S3A and D), confirming that the NMA modification was three to four-fold more potent than the MOE modification.

To determine whether the increased potency of the NMA-1 SSO observed in *SMN2* transgenic mice was due to greater accumulation in the CNS, we measured the amount of SSO in CNS tissues. The tissue concentration of the NMA-1 SSO was not higher than that of the MOE-1 SSO in the spinal cord and the brain (Supplementary Fig. S3B, C, E, and F). We also examined the relationship between the amount of the NMA-1 SSO in the CNS tissue and the degree of *SMN2* splicing correction. For each mouse that was dosed in Supplementary Fig. S3A and D, we measured SSO concentration in the spinal cord and the brain, and plotted these values against the degree of *SMN2* splicing correction in the spinal cord and the brain for the same mouse. We observed a strong correlation between SSO levels in CNS tissue and *SMN2* splicing correction. The half-maximal effective concentration (EC_50_) of the NMA-1 was ∼5 times lower than the MOE-1 SSO [0.3 µg/g versus 1.5 µg/g in the spinal cord (Supplementary Fig. S3B and C) and 1.1 µg/g versus 5.3 µg/g in the brain (Supplementary Fig. S3E and F)]. Therefore, even though both the MOE-1 and NMA-1 SSOs achieved similar CNS tissue concentrations, the NMA-1 SSO was approximately five-fold more active for *SMN2* splicing correction than the MOE-1 SSO, consistent with its higher potency on an ED_50_ basis.

Since the NMA-1 SSO showed enhanced potency in CNS tissues, we determined whether this was also the case in peripheral tissues. Increasing doses were administered subcutaneously to adult *SMN2* transgenic mice on days 1, 3, 5, and 7. Several peripheral tissues were analyzed for *SMN2* splicing correction 3 days after the last dose by RT-qPCR. In all tissues examined, both the NMA-1 and the MOE-1 SSOs resulted in a dose-dependent increase in *SMN2* splicing correction. The NMA-1 SSO was >2.5-fold more potent than the MOE-1 SSO for *SMN2* splicing correction (Supplementary Fig. S4). The potency of the NMA-1 SSO for *SMN2* splicing correction in peripheral tissues was further improved by ∼2–3 -fold when conjugated to palmitic acid (C16) (Supplementary Fig. S5). This suggests that alternative conjugation strategies for enhanced delivery to a variety of cell types and tissues [43] should be additive to the NMA modification. Additionally, the potency enhancement conferred by the NMA modification is distinct from C16-mediated protein binding-facilitated tissue uptake [43, 44].

Since the improved potency (on an ED_50_ basis) of the NMA SSO in *SMN2* splicing correction was consistent with a lower EC_50_, we hypothesized that the higher potency of the NMA SSO would be observed in cultured cells. Fibroblasts from an SMA patient were transfected by electroporation with increasing concentrations of the NMA-1 SSO or the MOE-1 SSO and *SMN2* splicing correction was analyzed after 24 h by RT-qPCR. The NMA-1 SSO was ~1.75-fold more potent than the MOE-1 SSO (*P *< .05; Supplementary Fig. S6; EC_50_ of NMA-1 = 0.08 µM versus MOE-1 = 0.14 µM). This suggests that the potency gain conferred by the NMA modification can be attributed to an intracellular mechanism. However, this interaction is not an increased binding affinity for the target site on the *SMN2* transcript since the *T*_m_ of the NMA-1 SSO is similar to the MOE-1 SSO (Supplementary Table S1).

### Optimizing the tolerability of the NMA-1 SSO

**Figure 2.**
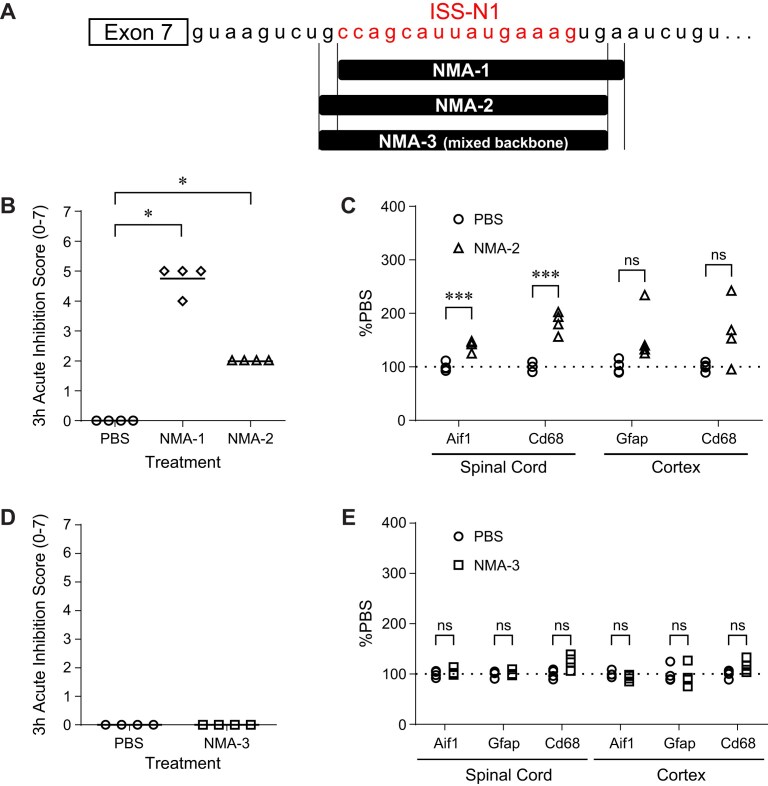
Sequence and chemistry optimization of NMA-1 SSO. (**A**) Schematic of positions of NMA SSOs on the *SMN2* pre-mRNA (partial sequence of intron 7 shown). (**B**) Acute inhibition scores in C57BL/6 mice 3 h after ICV bolus injection of PBS, 700 µg NMA-1, or NMA-2 SSOs. *N* = 4 mice per group; symbols represent individual animals; horizontal lines indicate the group mean. *P < .05, Mann–Whitney test versus PBS group. (**C**) RT-qPCR of astrocyte marker gene *Gfap*, microglia marker gene *Aif1*, and macrophage marker gene *Cd68* in spinal cord and cortex of C57BL/6 mice administered with PBS or 700 µg NMA-2 SSO. N = 4 mice per group; symbols represent individual animals. ***P < .001; ns, not significant, by multiple unpaired *t*-tests with Šídák correction. (**D**) Acute inhibition scores in C57BL/6 mice 3 h after ICV bolus injection of PBS or 700 µg NMA-3 SSO. *N* = 4 mice per group; symbols represent individual animals. (**E**) RT-qPCR of astrocyte marker gene *Gfap*, microglia marker gene *Aif1*, and macrophage gene *Cd68* in spinal cord and cortex of C57BL/6 mice administered with PBS or 700 µg NMA-3 SSO. *N* = 4 mice per group; symbols represent individual animals. ns, not significant by multiple unpaired *t*-tests with Šídák correction.

To evaluate the tolerability of the NMA-1 SSO, we delivered a high dose (700 µg) to the CNS of wild-type mice by ICV bolus injection. Bolus injection of high dose ASOs into the mouse CSF can lead to varying degrees of acute neuronal inhibition, and in the most severe cases, death [45]. To evaluate the tolerability of the NMA-1 SSO, we used a 7-point scale we previously established, in which higher scores reflect a more profound acute neuronal inhibition 3 h after SSO administration [45]. ICV bolus injection of the NMA-1 SSO resulted in an acute inhibition score of 5, manifested by the animal lying on its side without spontaneous movement after being transferred to a flat surface, while still responsive to a tail pinch. The NMA-1 SSO-mediated profound sedation was transient and the mice fully recovered after 24 h (Fig. 2A and B).

We and others have previously shown that the sequence of a gapmer ASO contributes significantly to acute neuronal inhibition [45–48]. To determine whether the acute inhibition caused by the NMA-1 SSO could be mitigated, we evaluated NMA-modified SSOs of different sequences that bound to sites within ISS-N1 at different positions than the NMA-1 SSO. An NMA-modified SSO (NMA-2) shifted by 1 bp (by loss of a 5′-T and gain of a 3′-C) relative to the NMA-1 SSO significantly improved the acute inhibition (score of 2) after a high dose ICV bolus injection to wild-type mice (Fig. 2A and B).

We next evaluated the long-term tolerability of the NMA-2 SSO in the mouse CNS by measuring glial marker gene (*Aif1, Gfap, Cd68*) expression 8 weeks following ICV bolus injection. High dose ICV bolus injection of the NMA-2 SSO increased the expression of the microglial markers *Aif1* and *Cd68*, which are associated with activated microglia and are indicative of neuroinflammation in the mouse CNS (Fig. 2C). The phosphorothioate (PS) backbone can influence protein-binding properties and tolerability of gapmer ASOs administered systemically [49–51]. To determine whether reducing PS content improves the long-term tolerability of SSOs in the CNS, we systematically substituted PS with phosphodiester (PO) linkages at different positions and ranked SSOs by glial marker expression levels. Among the 40 SSOs tested, 3 SSOs (SSO-19, SSO-22, SSO-28) elicited <140% of control levels of *Cd68* and/or *Gfap* (a marker of astrocyte activation) in the cortex and the spinal cord, with SSO-22 eliciting the least induction of *Cd68*. This suggests that strategic PS substitution with PO linkages at positions 2 and 4 from the 5′ end of the SSO (as in SSO-22) significantly reduced neuroinflammation associated with this SSO sequence (Supplementary Fig. S7). We then applied the same PO placement strategy to the shifted NMA-2 SSO and tested the SSO in wild-type mice. We confirmed that administration of this shifted NMA mixed backbone SSO (NMA-3) into the mouse CNS at a high dose did not induce a neuroinflammatory response (Fig. 2E). Furthermore, this PO placement further improved acute tolerability relative to the full PS-shifted NMA-2 SSO (Fig. 2D), consistent with previous findings that backbone PS content contributes to acute neuronal inhibition [45, 47, 48]. Altogether, the newly identified NMA-3 SSO has an excellent acute and delayed tolerability profile in mice.

### NMA-3 is more potent than nusinersen in human patient fibroblasts and in the CNS of human *SMN2* transgenic mice

**Figure 3.**
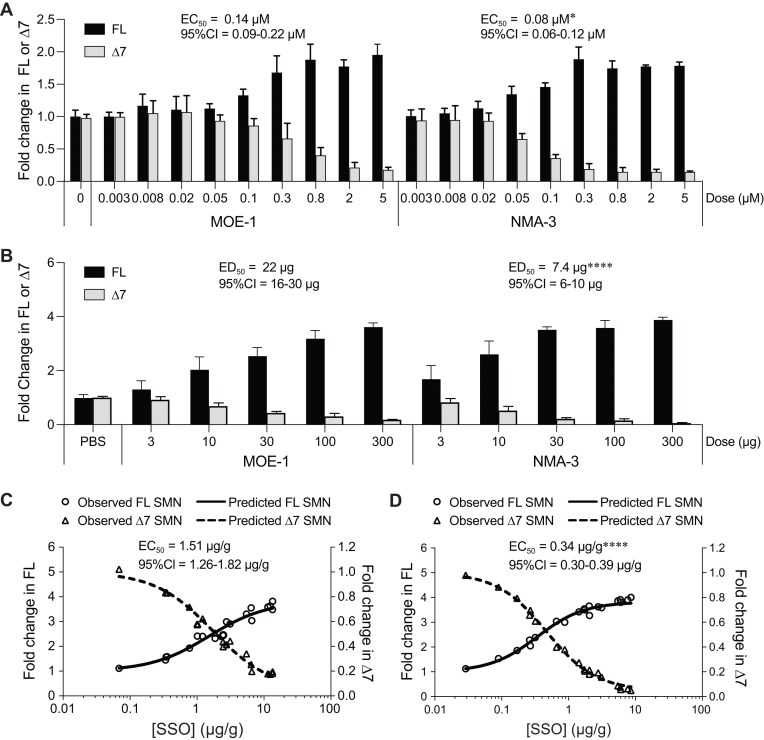
Potency for *SMN2* splicing correction of NMA-3 SSO compared with nusinersen (MOE-1). (**A**) RT-qPCR analysis of *SMN2* transcripts including exon 7 (FL) or skipping exon 7 (Δ7) in SMA patient fibroblasts treated with MOE-1 or NMA-3 SSOs. For each dose level, *N* = 3 replicate culture wells. Data are mean ± SD. The EC_50_ and 95% CI for FL correction are shown. Comparison of EC_50_ values between MOE-1 and NMA-3 was performed by nonlinear regression with extra sum–of–squares F test, *P < .05. (**B**) RT-qPCR analysis of FL or Δ7 *SMN2* transcripts in the spinal cord 14 days after ICV bolus injection of MOE-1 or NMA-3 SSOs in human *SMN2* transgenic mice. For each dose level, *N* = 4 mice per group. Data are mean ± SD. The ED_50_ and 95% CI are shown. Comparison of ED_50_ for *SMN2* FL correction between MOE-1 SSO and NMA-3 was performed by nonlinear regression with extra sum-of-squares F test, ****P < .0001. (**C**) Concentration of MOE-1 SSO in the spinal cord plotted against the level of FL or Δ7 *SMN2* transcripts measured in the spinal cord of each mouse dosed with MOE-1 SSO in panel (B). (**D**) Concentration of NMA-3 SSO in the spinal cord plotted against the level of FL or Δ7 *SMN2* transcripts measured in the spinal cord of each mouse dosed with NMA-3 SSO in panel (B). In panels (C) and (D), symbols represent individual animals. EC_50_ for *SMN2* FL correction and 95% CI are shown. Comparison of EC_50_ values between MOE-1 and NMA-3 was performed by nonlinear regression with extra sum-of-squares F test, ****P < .0001.

Having identified a well-tolerated NMA SSO (NMA-3), we first assessed its activity in fibroblasts from a patient with SMA. Fibroblasts were transfected by electroporation with increasing concentrations of the NMA-3 SSO or nusinersen (MOE-1) and *SMN2* splicing correction was analyzed after 24 h by RT-qPCR. Like NMA-1 in patient fibroblasts (Supplementary Fig. S6), NMA-3 SSO produced a dose-dependent increase in *SMN2* splicing correction, with an EC_50_ of 0.08 µM. NMA-3 was ∼1.75-fold more potent than the MOE-1 SSO, which had an EC_50_ of 0.14 µM (Fig. 3A).

Next, we assessed the activity of the NMA-3 SSO in human *SMN2* transgenic mice. Increasing doses of the NMA-3 and MOE-1 SSOs were administered as a single ICV bolus injection and CNS tissues were analyzed for *SMN2* splicing 2 weeks after the injection by RT-qPCR. We observed a dose-dependent increase in *SMN2* splicing correction with the NMA-3 SSO with an ED_50_ in the spinal cord of 7.4 µg (Fig. 3B) and 17 µg in the brain (Supplementary Fig. S8A). The NMA-3 SSO was three-fold more potent than the MOE-1 SSO. The MOE-1 SSO had an ED_50_ of 22 µg for exon 7 inclusion in the spinal cord (Fig. 3B) and 32 µg in the brain (Supplementary Fig. S8A). As expected, the NMA-3 SSO was also more potent than the MOE-1 SSO when the SSO levels in the CNS tissue were compared to the extent of *SMN2* splicing correction in each animal. The EC_50_ in the spinal cord was 0.34 µg/g for the NMA-3 SSO and was 1.51 µg/g for the MOE-1 SSO (Fig. 3C and D). The EC_50_ in the brain was 2.12 µg/g for the NMA-3 SSO and was 5.35 µg/g for the MOE-1 SSO (Supplementary Fig. S8B and C). Additionally, the improved potency of the NMA-3 SSO was attributable to the NMA modification and not its sequence or backbone composition (Supplementary Fig. S9).

### The NMA-3 SSO has a long duration of action in the CNS of human *SMN2* transgenic mice

**Figure 4.**
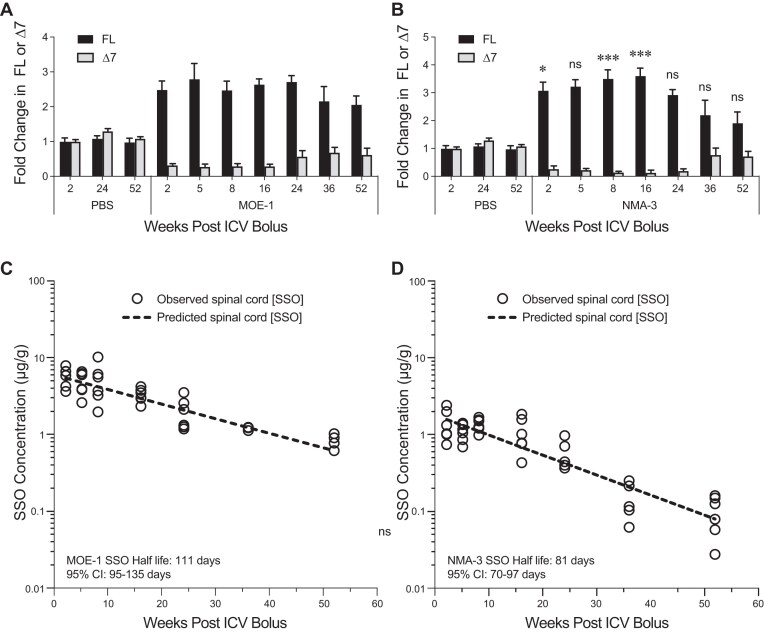
Duration of action and half-life of NMA-3 SSO compared with nusinersen (MOE-1) in the spinal cord of human *SMN2* transgenic mice. (**A**) RT-qPCR analysis of FL or Δ7 *SMN2* transcripts in the spinal cord at the indicated timepoints after ICV bolus injection of PBS or 100 µg of MOE-1 in the human *SMN2* transgenic mice. *N* = 5–6 mice per group per time point. Data are mean ± SD. (**B**) Same as panel (A), except mice received 50 µg of NMA-3 SSOs instead of MOE-1. *N* = 5–6 mice per group per time point. Data are mean ± SD. Differences between MOE-1 and NMA-3 groups for FL transcripts were assessed by two–way ANOVA with post–hoc Šídák’s multiple comparisons test at each time point, *P < .05, ***P < .001. (**C**) MOE-1 SSO spinal cord tissue concentration versus time after a single 100-µg ICV bolus injection. (**D**) NMA-3 SSO spinal cord tissue concentration versus time after a single 50-µg ICV bolus injection. In panels (C) and (D), *N* = 5–6 mice per time point; symbols represent individual animals.

Previously we demonstrated that in human *SMN2* transgenic mice nusinersen (MOE-1) has a long duration of action with sustained *SMN2* splicing correction for over 52 weeks after a single ICV bolus injection [34]. However, the NMA modification and PO modifications present in the NMA-3 SSO could have an impact on duration. Here, we determined the duration of action of the NMA-3 SSO in human SMN2 transgenic mice and directly compared it with that of the MOE-1 SSO. To account for the enhanced potency of the NMA-3 SSO, we performed a single ICV bolus injection of 50 µg for the NMA-3 SSO and 100 µg for the MOE-1 SSO. The level of *SMN2* splicing correction was measured at various time points after the ICV bolus injection up to 52 weeks. The NMA-3 SSO displayed sustained *SMN2* splicing correction in the spinal cord and the brain (Fig. 4 and Supplementary Fig. S10), comparable to what was observed with the MOE-1 SSO. The long-lasting *SMN2* splicing correction observed with the NMA-3 SSO was attributable to its long half-life in the mouse CNS tissue (81 days in the spinal cord and 196 days in the brain).

### Enhanced potency of an NMA-modified *SCN1A* SSO

**Figure 5.**
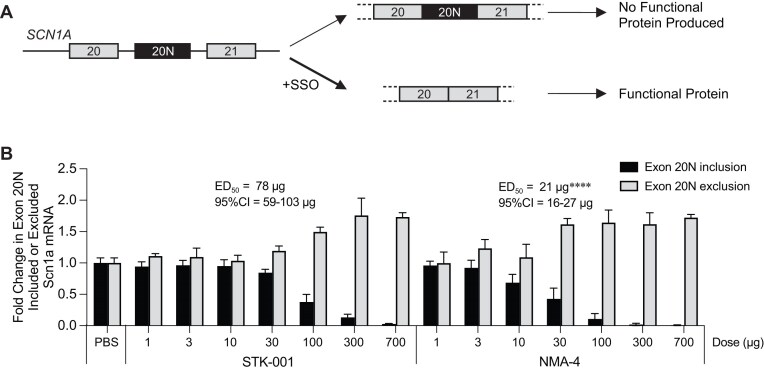
Potency comparison of MOE and NMA SSOs for splicing modulation of *SCN1A* mRNA in the brain of mice. (**A**) Schematic diagram of naturally occurring productive versus unproductive alternative splicing of *SCN1A* pre-mRNA. Exon 20N-excluded transcript produces functional Nav1.1 protein whereas exon 20N-included transcript undergoes NMD resulting in no functional protein production. SSO promotes exon 20N skipping to restore functional protein production. (**B**) RT-qPCR analysis of mouse *Scn1a* transcripts including exon 20N (nonproductive transcript) or skipping exon 20N (productive transcript) in C57BL/6 mice after ICV injection of different doses of STK-001 or NMA-4 SSOs. For each dose level, *N* = 4 mice, except *N* = 8 for PBS group and *N* = 2 for STK-001 700 µg group. Data are mean ± SD. The ED_50_ for exon 20N inclusion and 95% CI are shown. Comparison of EC_50_ values between STK-001 versus NMA-4 SSO was performed by nonlinear regression with extra sum-of-squares F test, ****P < .0001.

A therapeutic strategy that is being pursued for the treatment of Dravet syndrome is to use an SSO to promote skipping of exon 20N in transcripts produced from the *SCN1A* gene (Fig. 5A). This strategy has been shown to increase the levels of *SCN1A*, which is reduced in Dravet syndrome patients [26, 27]. The SSO previously used to induce skipping of *SCN1A* exon 20N is an 18-mer uniformly modified with MOE referred to as STK-001 [26, 27]. To determine whether the NMA modification could also enhance splicing correction of SCN1A, we tested an NMA-modified SSO targeting exon 20N with the same sequence as STK-001. We administered increasing doses of the NMA version of STK-001 (here called NMA-4) or STK-001 to wild-type mice via an ICV bolus injection. Brain tissue was analyzed for *SCN1A* transcripts with or without exon 20N by RT-qPCR 4 weeks post-injection. As expected, STK-001 showed a dose-dependent reduction in transcripts containing exon 20N, and an increase in transcripts that exclude exon 20N. The ED_50_ for splicing correction in the brain was 78 µg for STK-001 and the NMA-4 SSO was 3.5-fold more potent with an ED_50_ of 21 µg (Fig. 5B).

## Discussion

Our work demonstrates a novel use of NMA chemistry in SSOs, resulting in substantially improved activity. NMA-modified SSOs are more potent than MOE-modified SSOs for correcting splicing of both *SMN2* exon 7 and SCN1A exon 20N. We believe that the NMA modification will also exhibit higher potency for additional, well-established therapeutic targets where splicing modulation is required.

We demonstrated in fibroblasts from a patient with SMA and in human *SMN2* transgenic mice that an NMA-modified SSO is significantly more potent for *SMN2* splicing correction than a MOE-modified SSO. Additionally, we identified an NMA-modified human candidate SSO (NMA-3) that is three to four-fold more potent than nusinersen in our experimental models. Following extensive structure-activity relationship studies, we showed that NMA-3 is well tolerated in mice. Our newly identified NMA SSO also exhibits a long duration of action in human *SMN2* transgenic mice, which is similar to nusinersen. The current clinically approved dosing schedule for nusinersen is four loading doses in the first 2 months, followed by dosing every 4 months. The higher potency of the NMA-3 SSO should allow for similar clinical efficacy to nusinersen but with less frequent dosing, thereby reducing the burden of repeated lumbar punctures for patients and caregivers. In fact, NMA-3 (salanersen) is currently being evaluated in a Phase 3 clinical trial (NCT07221669) in SMA patients with a once-yearly dosing regimen.

The exact mechanism by which the NMA modification modulates splicing more potently than the MOE modification is not known. Current evidence indicates that the NMA modification does not improve potency by increasing CNS tissue accumulation, since it accumulates to the same extent as the MOE-modified SSO (Fig. 3C and D). The fact that the NMA SSO has a lower EC_50_ for *SMN2* splicing correction in CNS tissues of human *SMN2* transgenic mice than the MOE SSO and that the NMA SSO is more potent than the MOE SSO in cell culture when delivered by electroporation (Fig. 3A) suggests that the NMA modification is providing a benefit intracellularly. However, this is not due to an increased affinity for binding to its target site on the RNA since the *T*_m_ of NMA-modified SSOs is comparable to MOE-modified SSOs (Supplementary Table S1). Potential contributors to the improved activity conferred by NMA modification include improved release from endosomes [52, 53], better trafficking to the nucleus and/or the site of transcription on chromatin where splicing takes place [35, 54], or interactions with splicing factors or RNA binding proteins after the NMA SSO has hybridized to its target site as has previously been shown for 2′-F-modified SSOs [35]. More work is needed to determine how the NMA chemistry enhances the potency of SSOs.

## Supplementary Material

gkag484_Supplemental_Files

## Data Availability

All data supporting the findings of this study are available within the article and its Supplementary data. Raw qPCR and bioanalytical datasets are available from the corresponding author upon reasonable request.
